# Exploring the role of patient activity in the clinical decision-making processes of health care practitioners working in hospital care: A qualitative study

**DOI:** 10.1177/02692155251393557

**Published:** 2025-11-12

**Authors:** Carlos de Miguel Llorente, Miriam Van Der Velde, Ireen Scheffers, Cindy Veenhof, Karin Valkenet

**Affiliations:** 1 Department of Rehabilitation, Physiotherapy Science and Sport, 526115 University Medical Center Utrecht, Utrecht, The Netherlands; 2 Research Center for Healthy and Sustainable Living, Research group Innovation of Movement Care, 8119 University of Applied Sciences Utrecht, Utrecht, The Netherlands; 3 Department of Hematology, 8124 University Medical Center Utrecht, Utrecht, The Netherlands

**Keywords:** Patient activity, physical activity, clinical decision-making

## Abstract

**Objective:**

This study aims to explore the role of patient activity in the clinical decision-making processes of various health care practitioners working in hospital care.

**Design:**

Qualitative research study.

**Setting:**

The study was conducted in the hospital care setting of UMC Utrecht, the Netherlands.

**Participants:**

Health Care Practitioners working in the hospital setting were selected through purposive sampling. Doctors, nurses, and physiotherapists were included if their professional activity centered on inpatient care, they had worked in the hospital for over four weeks and were not students in the department.

**Main measures:**

Data were collected through observations and interviews. Interview recordings were transcribed, and both the interview transcripts and observation fieldnotes were coded and analyzed using reflexive thematic analysis, following Braun and Clarke's approach. Themes were iteratively reviewed and refined, leading to the development of core themes, which were constructed by synthesizing subthemes and examining their interrelationships.

**Results:**

The study involved forty-two participants working at UMC Utrecht. Four core themes were identified: 1) Patient activity influences clinical decision-making, 2) clinical decision-making influences patient activity, 3) multidisciplinary dynamics influence how patient activity is valued in clinical decisions, and 4) limited use of objective measurements influences how patient activity informs clinical decisions.

**Conclusion:**

This study explores the complex interaction between clinical decision-making and patient activity and how patient activity is integrated from a multidisciplinary perspective in the hospital setting. The results highlight the importance of interdisciplinary communication, barriers and facilitators for improved decision-making and examines both implicit and explicit processes involved.

## Introduction

Patient activity during hospital stay has garnered significant attention in the past few years.^1–5^ Higher levels of physical activity play an important role in reducing functional decline and minimizing muscular, lung, and hematological side effects associated with hospitalization.^3,6–8^ Hence, there has been an increase in both research and implementation of interventions to promote physical activity and reduce sedentary behavior during hospital stay.^9–11^

Although health care practitioners work collaboratively to reduce these adverse effects of admission, addressing this deeply rooted sedentary culture within hospitals presents a strong challenge.^12–14^ Health care practitioners are aware of the benefits of higher levels of physical activity; however, despite this awareness, they face challenges in integrating physical activity to improve patient care.^15–17^ As a result, the hospital environment often perpetuates a culture of immobility, where patient activity is frequently deprioritized.^11,12,14^ Therefore, there is a need to find ways to improve the prioritization of patient activity in hospital care.

In daily care practice, health care practitioners across various specialties implicitly rely on patient activity as an indicator of disease progression and recovery after a surgical procedure.^18–20^ At the same time, clinical decisions—such as prescribing medications, adjusting recovery protocols, or assessing readiness for surgery—have been shown to be influenced by patient activity.^21–24^ This indicates that patient activity already plays a larger role in daily hospital care than many health care practitioners are aware of.

Making patient activity a more explicit factor in clinical decisions could increase its visibility and priority in daily hospital care. However, it remains unclear to what extent patient activity is actually considered in routine clinical practice. Therefore, this study uses a reflexive thematic analysis to understand the role of patient activity in the clinical decision-making of different health care practitioners working in hospital care.

## Methods

### Study design

This qualitative study employed observations and semi-structured interviews. Using two methods of data collection allowed us to capture both what practitioners did in their daily work (observations) and how they explained and reflected on these practices and decision-making processes (interviews).^25^ Data were analyzed using inductive reflexive thematic analysis, following Braun and Clarke,^26^ within a constructivist perspective. In this approach, knowledge was developed from interpreting both what practitioners did (observations) and how they explained those actions (interviews), within their social and professional context. In qualitative research, inductive analysis involves generating patterns and themes directly from the data, rather than testing or applying existing theories or framework.^27^ Inductive analysis was chosen because this study explored an under-researched area, allowing themes to emerge directly from the data rather than from predefined categories. This ensured that the resulting themes reflected both unexpected insights and the complexity of practitioners’ decision-making.^26^

### Setting and sampling

The study was conducted at University Medical Center Utrecht, The Netherlands. We included doctors, nurses, and physiotherapists as these specialisms are primarily responsible for patient activity during hospital stay. Participants were eligible if they primarily worked on a ward providing inpatient care, they had been working in the hospital for more than 4 weeks and they were not students in the department. To ensure diverse perspectives, participants were purposively sampled from multiple wards and varied in specialty, role, and seniority. Participants were recruited through face-to-face contact and email. After receiving study information, they were invited to voluntarily participate in interviews and/or observations. Recruitment began with physiotherapists, as the researcher had an established professional relationship with this group. These initial contacts then facilitated access to nurses and doctors.

### Data collection

Data collection consisted of two parts: observations and semi-structured interviews. Participants were either observed, interviewed or both.

Naturalistic behavioral observations of healthcare practitioners were conducted during their routine clinical practice. Each participant was observed once, for a continuous period of 1–2 h. Observations were scheduled at different times of day to capture a range of clinical contexts and activity patterns. To enhance understanding of observed behaviors, the observer could engage in brief conversations with participants during the observation period. Clarifying questions were asked when specific actions or decisions were not immediately clear to the researcher. Observations focused exclusively on the recruited participants; no identifiable information of non-participants was recorded, and observations were paused if anyone objected. A data observation tool was used which combined predefined interactions and open information (Supplementary 1). The predefined categories (e.g., how many times the health care practitioner asked colleagues about patient activity, and how many clinical decisions were made based on patient activity) were developed based on earlier behavioral mapping studies and literature on patient activity.^9,12,15,28^ In addition, there was space for fieldnotes to capture contextual details beyond the predefined categories.

Semi-structured interviews were conducted to complement the information from the observations. Interview participants included both previously observed individuals and new ones. For those who had been observed, the interviews referred to their observed actions. Each participant was interviewed separately. Interview guides were developed based on prior research on patient activity,^11,14,28^ insights from the observational data, and a practice-oriented focus to ensure relevance to clinical work (see Supplementary 2). Example questions included: “How do you observe and interpret patients’ activity during clinical encounters?” and “How do you exchange patient activity information with other HCP?”. The guide was used flexibly, allowing participants to elaborate on their own experiences and perspectives. An iterative process of code identification was used meaning that each interview was affected by the previous one. All the interviews were in-person and were estimated to have 30 min of duration. Every interview was recorded using a voice recorder to be transcribed later and stored on a locally secured drive.

### Data analysis

Observation notes, recorded on the data observation form, were expanded into fieldnotes. Interview recordings were transcribed using Microsoft 365's built-in transcription feature and manually reviewed for accuracy. Both observation and interview data were coded manually in NVivo. The coding process began with inductive line-by-line coding to identify meaningful units of text related to patient activity and clinical decision-making, resulting in a large set of initial codes (CDM). These were then compared and merged through focused coding into broader categories, which were iteratively refined into subthemes (CDM, MV). Finally, subthemes were reviewed and synthesized into overarching themes until consensus van reached (CDM, KV).

Theoretical saturation was assessed for both observations and semi-structured interviews. Data collection continued until sufficient depth and diversity of perspectives had been captured and no substantially new patterns or codes were identified. Once saturation of the observational data was reached, observation data collection ceased, and interviews were initiated. These interviews were designed based on the insights and preliminary codes generated from the observations. Interview data collection continued until the appearance of new codes declined, and further interviews did not generate additional theoretical insights. This process of reaching theoretical saturation is embedded within reflexive thematic analysis.^29^

### Ethical considerations

This study did not fall under the scope of the Dutch Medical Research Involving Human Subjects Act (WMO). It therefore did not require approval from an accredited medical ethics committee in The Netherlands, However, in the University Medical Center Utrecht, an independent quality check was conducted to ensure compliance with legislation and regulations (regarding Informed Consent procedure, data management, privacy aspects and legal aspects). Consent was given after review of the protocol (Protocol number 24U-0064). All the participants were clear and aware on the purpose and role of the observer and interviewer and the research project. All of them provided written informed consent before the observations and the interviews, and they were told that they could withdraw from the study at any time. All data concerning participants information were pseudonymized and then securely stored.

### Quality and rigor

Several measures were taken to ensure rigor. Three observations were conducted with two observers (CDM and KV) to enhance accuracy. Two researchers (CDM and MV) independently coded transcripts to verify code identification. Memos were kept as part of an audit trail. Focus coding and theme development were carried out by two researchers (CDM and KV), with one coder blind to the initial codes. Data triangulation was achieved through both observations and interviews. All team members had prior qualitative research experience and engaged in ongoing reflexivity. Further details on the research team and recruitment are provided in Supplementary 3.

## Results

Data was collected between January 2024 and August 2024. Data was gathered from 42 participants (12 doctors, 14 physiotherapists and 16 nurses). Twenty-one participants had been working as health care practitioner in their ward for less than five years (Junior), while the other 21 had been working for more than five years (Senior). In total 16 participants were observed, 21 interviewed, and five were both observed and interviewed. Interviews had a mean length of 20.1 (4.33) minutes while observations had a mean length of 73.2 (25.38) minutes. Characteristics and distribution of the participants are shown in Table 1.

**Table 1.** Participant characteristics by profession, clinical experience, and ward assignment (frequencies and percentages included).

Category	Label	Total
Health Care Practitioners		n. %
	Doctor	12 (28.57)
	Nurse	16 (38.09)
	Physiotherapist	14(33.33)
Seniority		n, %
	Junior	21 (50)
	Senior	21(50)
Ward		n, %
	Cardiology	4 (9.52)
	Gastroenterology	1(2.38)
	Geriatrics	3(7.14)
	Hematology	8 (19.04)
	Intensive Care Unit	2(4.76)
	Internal Medicine	4(9.52)
	Pneumology	4(9.52)
	Neurological Surgery	2(4.76)
	Neurology	7(16.66)
	Oncology	3(7.14)
	Traumatology	2(4.76)
	Rehabilitation Medicine	1(2.38)

Thirteen subthemes were identified which were allocated into four core themes: i) Patient activity influences clinical decision-making, ii) Clinical decision-making influences patient activity, iii) Multidisciplinary dynamics influence how patient activity is valued in clinical decisions and iv) Limited use of objective measurements influences how patient activity informs clinical decisions.

**Figure 1.**
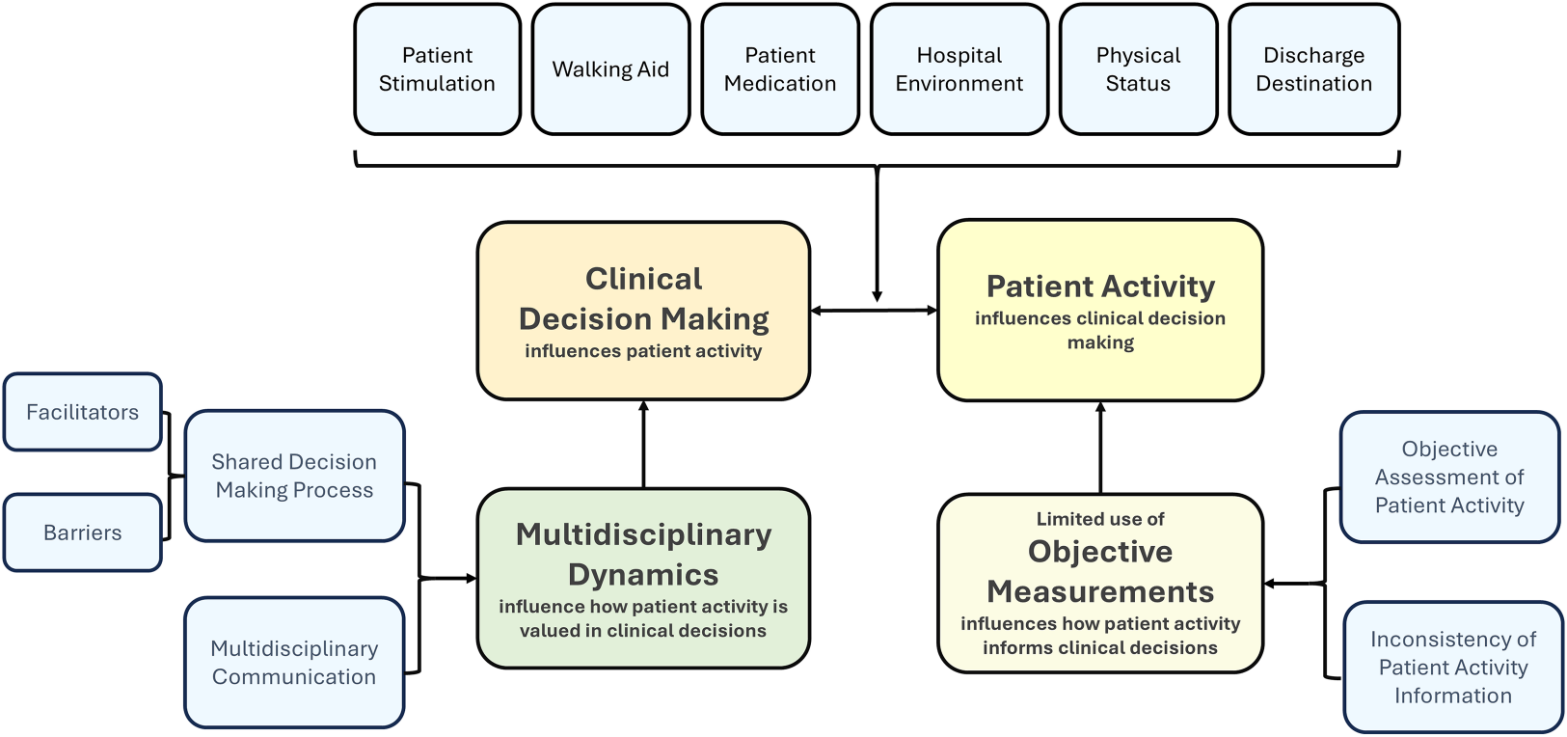
Matrix illustrating the interplay between clinical decision-making and patient activity: this figure presents a conceptual matrix showing the relationships between clinical decision-making (CDM) and patient activity (PA), including major themes, subthemes, and the contributing or influencing factors identified in the analysis.

These themes highlighted various aspects of how patient activity influences decision-making, multidisciplinary management and the barriers and facilitators of decision-making. An illustration of the themes and subthemes, working together and interacting with each other is shown in Figure 1. It depicts a matrix of interrelationships among these themes, rather than describing a linear process. Each of the subthemes are available in Table 2. Moreover, Table 3 provides an illustrative example of the analytic process, showing how raw data were coded and subsequently organized into subthemes and main themes. Representative participant quotes illustrating each subtheme within the main themes is shown in Supplementary 4.

**Table 2.** Subthemes within the main themes.

Themes	Subthemes
1.Patient activity influences clinical decision making	Decisions on discharge destination
	Decisions on patient medication
	Decisions on walking aid
	Decisions based on physical status
2. Clinical decision-making influences patient activity	Medication on patient activity
	Patient stimulation on patient activity
	Hospital environment on patient activity
3. Multidisciplinary dynamics influence how patient activity is valued in clinical decisions	Multidisciplinary communication
	Facilitators for shared decision-making
	Barriers for shared decision-making
	Shared decision-making process
4. Limited use of objective measurements influences how patient activity informs clinical decisions	Inconsistency of patient activity information
	Objective assessment of patient activity

**Table 3.** Example of the coding process from raw data to main themes.

Raw Data (from observations or interviews)	Code	Subthemes	Themes
“I think it is very important to have a discussion with the patient, their family, and close contacts to explore what matters most to the patient and to agree on the goals of treatment.” (Geriatric doctor, Interview)	Involving family in decision making	Shared decision-making process.	Multidisciplinary dynamics influence how patient activity is valued in clinical decisions
“When decisions are made about major surgeries, other specialties often consult us to consider whether the treatment is still proportional for the patient or whether it may be too much.” (Internal medicine doctor, Interview)	Assessing patients’ patient activity to decide if they are suitable for surgery.	Decisions based on physical status	Patient activity influences clinical decision making
“During the observation, the physiotherapist explained that a patient was able to walk safely with a walker but tired easily. The doctor then added that, from a medical perspective, treatment was complete, and that the remaining issues concerned mobility and preparing the patient for discharge.” (Physiotherapist, Observation multidisciplinary team meeting)	Physical recovery lags behind medical recovery	Decisions on discharge destination	Patient activity influences clinical decision making
“Well, at the moment I don’t think we measure patient activity on this ward. On the previous ward where I worked, we did measure it — we scored it and had better coordination with the physiotherapists and dietitians, who were also more experienced in doing these assessments. Here, I think we approach it in a less structured way.” (Gastroenterology doctor, Interview)	Having more objective measurement would lead to better results.	Inconsistency of patient activity information	Limited use of objective measurements influences how patient activity informs clinical decisions

### Core themes

#### Patient activity influences clinical decision-making

We identified several clinical decisions during hospital stay that were influenced by the patient's activity or functional status, defined as the ability to perform daily activities necessary to meet basic needs.

First, clinical decisions regarding post-discharge destination. Both observations and interviews highlighted that there were no clear or standardized thresholds to determine whether a patient was ready for discharge. Observations of multidisciplinary meetings showed that factors such as the type of home, and whether the patient lived alone, shaped how practitioners defined the level of activity and functional status required for a safe discharge. In interviews with doctors caring for older patients, it was frequently mentioned that patients were often medically ready for discharge but lacked the necessary independence to return home. When making discharge decisions, doctors considered patient activity a key factor, as patients might not be ready to return home if their living environment involved barriers such as stairs or the absence of an elevator.

Second, certain pharmacological decisions were also influenced by patients’ activity. Thrombosis prophylaxis was a frequently cited example. Doctors and nurses noted in the interviews that when patients were insufficiently active, prophylaxis would be initiated, while increased patient activity often led to its withdrawal. These decisions were not based on explicit numerical thresholds but rather on whether the patient was perceived to be more active.

“We must give medication when patients are immobilized to prevent thrombosis….so that is the first thing, but that does not really depend on the physiotherapist… That is just if the patient is immobilized. So, we are giving him medication to prevent complications from staying in bed…” [Doctor, Geriatrics, Interview, P26]

Third, patient activity and functional status also influenced decisions about the use of walking aids or independent mobility. Observations showed that these decisions were commonly made directly on the ward by mainly nurses and physiotherapists, based on the quality and quantity of patients’ gait. In interviews, doctors confirmed this division of responsibility, noting that they typically delegated such decisions to them. When asked whether standard criteria guided their choices, nurses and physiotherapists explained that decisions relied on intuition, subjective interpretation, and professional experience rather than on consensus or formal guidelines. Underlying these decisions were concerns about patient safety and fall risk. Across interviews, a recurrent dilemma was the balance between safety and activity: doctors, nurses, and physiotherapists often feared that encouraging activity could increase walking time and elevate the risk of falls. In most cases, practitioners choose to prioritize safety over activity. Patient activity was thus used as a key indicator of safety in decision making. For example, if patients appeared at high risk of falling, additional support such as walking aids or rehabilitation was recommended. In this way, concerns about falls made patient activity a central factor in decisions about ongoing care and discharge readiness.

“They (nurses and physiotherapists) see the patients all the time. I believe them more than myself, so that's why we also ask for their opinions during the multidisciplinary meetings, and I can say they are really good in saying what is the current condition, strength, balance, and what advice they have for using a walker or other things. (to support gait) …” [Doctor, Gastroenterology, Interview, P19]

Fourth and final, several clinical decisions were made based on the patient's physical status, defined as the overall condition of an individual's body, including aspects like body composition, strength, and endurance. These decisions covered deciding chemotherapy treatment, use of mechanical ventilation or if a patient was prepared to receive surgery. Doctors and nurses working in surgical care noted during interviews that when patients were physically active, doctors often interpreted this as an indicator of overall good condition. If a patient was unable to move and showed poor physical status, they might not be considered ready for certain treatments, as their frailty could increase the likelihood of poorer surgical outcomes.

“We would never give a patient chemotherapy for a newly discovered cancer, which has spread everywhere when he or she is not able to walk, for example when it's a nursing home patient in the bed…” [Doctor, Geriatrics, Interview, P37].

#### Clinical decision-making influences patient activity

Just as patient activity influenced clinical decision-making, certain clinical or hospital decisions can, in turn, affect patients’ activity. The interaction between clinical decision-making and patient activity is illustrated in Figure 1. This relationship follows a feedback-feedforward dynamic, where each factor influences and shapes the other.

First, clinical decisions regarding medication can affect patient activity. Pharmacological guidelines after surgical procedures or medication required to treat the medical reason the patient was admitted for, can affect the patient's activity. An example of this is the effect of administering opioid pain medication on patient activity. Nurses and physiotherapists explained during interviews that if the patient is receiving a complex treatment that involves intense pain, the doctors will decide to use analgesics or opioids to alleviate their pain. The excess of opioids will make the patient more somnolent and therefore reduce their energy and desire to move. Hence, the patient will move less.

“It is possible when patients get too much medication like morphine…they can be very sleepy, and they do not need that much (medication). Then the patient is more somnolent and does not want to move…” [Physiotherapist, Traumatology, Interview, P16]

Second, ward observations of nurses demonstrated the importance of patient stimulation and how it affected patient activity. When nurses noticed that a patient was not moving, they often responded by encouraging the patient to be active or by notifying a physiotherapist. Conversely, when no clinical action was taken, patients’ activity was often limited by a lack of stimulation or guidance, which could lead to continued lack of patient activity. Observations of nurses, later confirmed in interviews, showed that they relied on guidelines and protocols to promote patient activity. These included post-surgery mobilization protocols and benchmarks for independent movement. By following such guidelines and actively encouraging activity, nurses made clinical decisions that directly shaped patient activity. Therefore, when nurses applied these guidelines or decisions, patient activity was directly affected.

Finally, non-clinical factors, such as the hospital environment and overall hospital setting, also influenced patient activity. Although these were not clinical decisions per se, they reflected broader organizational choices. In interviews, nurses with experience across different hospitals/wards explained that changes to the physical environment, such as creating more space for exercising or altering the layout of ward areas, could significantly affect patient willingness to move and therefore patient activity.

#### Multidisciplinary dynamics influence how patient activity is valued in clinical decisions

Some decisions were made jointly by doctors and other health care practitioners. These included determining discharge location, initiating, or stopping physiotherapy, deciding on the use of walking aids or determining when patients could walk independently. Although some of these decisions were described in Theme 1(“Patient activity influences clinical decision making”), they also emerged as clear examples of collaborative decision-making in both observations and interviews. Therefore, we also included them in this theme. Observations showed that such joint decisions were made not for every patient but mainly for more complex cases, such as those with extended hospital stays or those at substantial risk. Decisions about whether a patient was ready to return home or required transfer to a nursing home were taken with input from practitioners directly involved in the patient's care.

The main multidisciplinary interactions where patient activity was discussed included morning rounds, multidisciplinary meetings, and informal encounters on the ward. First, during observations of morning rounds, discussions of patient activity mainly focused on whether the patient was moving or not. When discharge was approaching, the conversation shifted to the discharge destination and whether the home environment posed barriers, such as a staircase, that required the patient to have sufficient functional status for a safe discharge. Second, during observations of multidisciplinary meetings, practitioners discussed patients’ general progress, the use of walking aids, and the initiation or modification of physiotherapy sessions. In these discussions, the input of nurses and physiotherapists was especially valued, as they were most familiar with patients’ daily activity and progress. Finally, observations highlighted the importance of informal encounters on the ward as a source of communication. In these interactions, nurses played a leading role. Because they spent the most time with patients, nurses were often the first to be approached with questions from doctors and physiotherapists, a point they also confirmed in interviews. Multidisciplinary interactions, the matters addressed, and the health care practitioners involved are further shown in Supplementary 5.

“So, I create an overview of what was the patient able to do before the hospital, what the patient can do now and then what do I expect that the patient wants to do afterwards… the patient needs to go back to where he was…” [Doctor, Rehabilitation Medicine, Interview, P40]

In specific wards such as neurology, traumatology, and geriatrics, the rehabilitation medicine physician was frequently consulted for complex patients. When asked directly about their role during the interviews, the rehabilitation physician explained that they contributed to decisions concerning patient activity, such as whether a walking aid was needed or when the patient could begin walking after surgery. Moreover, certain actions and behaviors of health care practitioners acted as either barriers or facilitators to multidisciplinary decisions related to patient activity. These included decisions about the use of walking aids, the setting of activity goals, and the level of independence given to a patient to walk. As for facilitators, participants emphasized in interviews that multidisciplinary communication and shared goal setting with other practitioners improved decision-making. Nurses highlighted the importance of being aligned with colleagues and sharing common standards for patient activity, which facilitated the early identification of patients who were not moving as expected. They emphasized that many patients remained inactive for long periods, which they viewed as unnecessary and potentially harmful. In addition, inactivity was observed more often than excessive activity, and nurses therefore believed that reducing inactivity should be prioritized. Most barriers to multidisciplinary decision-making related to unclear professional boundaries, particularly regarding who was responsible for assessing, reporting, and discussing patient activity with other practitioners.

During a ward round, when the team discussed a patient's mobility, the doctor asked about the patient's movement. The nurse explained that the patient was using a walker, moving around the room independently, and had begun going to the bathroom alone, emphasizing that no falls had occurred. [Nurse and Doctor interaction, Geriatrics, Observation, P14].

#### Limited use of objective measurements influences how patient activity informs clinical decisions

During the interviews, most of the participants agreed that one of the main barriers to integrate patient activity in their decision-making process was the challenge of having objective measurements of patient activity.

Observations of the nurses showed that in some wards, the quality of movement (quality of gait, self confidence of the patient, etc.) was prioritized, while in others the distance walked, or the duration of movement was more important. Certain wards meticulously recorded any change in patient position in the electronic health record, whereas others rarely documented changes of patients’ activity. This inconsistency in recording patient activity information was evident across wards and in most of them, none of the health care practitioners used objective tools to record it.

During observations of interdisciplinary encounters, it was rare to see shared, objective information about patient activity. In the interviews, participants noted that this lack of objective measurement was a major impediment to accurately assessing and integrating patient activity into decision-making. Participants who worked in the few wards where movement monitors (activity trackers) were used (hematology and geriatrics) explained during the interviews that patients enjoyed the fact of visualizing the amount of physical time they had per day. Unfortunately, only ten participants reported to have used the data of the movement monitors to take clinical decisions. They admitted lacking the sufficient knowledge to use and interpret these data. Participants also mentioned during the interviews that in the future, this data could help them to have more specific measurements and therefore more awareness of patients’ activity.

“I think that this will at least allow us to map out how much someone moves. So that's definitely a good measuring tool. And on the basis of that, you can of course talk to someone about whether that is going well or whether it could be improved…” [Nurse, Internal Medicine, Interview, P30]

## Discussion

This study investigated how health care practitioners in the hospital setting use patient activity in their clinical decision-making processes. Our findings show that patient activity and decision-making are linked in both directions: clinical decisions shape how patients move during admission and recovery, while patient activity in turn informs decisions about discharge planning, medication, and surgical eligibility. Clinicians often interpreted patient activity based on intuition and experience. Having clearer, structured ways to evaluate patient activity could support more consistent integration of patient activity into clinical decisions. Finally, the absence of a shared language across wards limited the effective exchange of activity-related information. Standardized, objective terminology and measurements could help strengthen interprofessional communication and improve how patient activity is integrated into clinical decisions.

It is complex to determine the precise influence of patient activity on clinical decision-making, as patient activity was often considered in an implicit rather than explicit manner. For instance, practitioners never reported using fixed criteria such as a minimum number of minutes walked, or a certain distance walked as a basis for clinical decisions. Instead, activity was incorporated more implicitly, with practitioners relying on their experience to judge whether patients with low activity or poor functional status would benefit from treatments, surgeries, or rehabilitation. In this sense, patient activity informed decisions, but rarely through standardized or explicitly articulated criteria. Physiotherapists have been shown to use explicit decision-making regarding patient activity, as illustrated in the study by Christensen et al.^30^ In that study, objective assessment tools were used to guide clinical reasoning, set goals, and establish a prognosis. This provided physiotherapists a more comprehensive understanding of patient's physical condition, leading to improved assessment. Still, the assessments were always being complemented by intuitive evaluations of patient's activity and functional status. In contrast, in our study, although physiotherapists were in some instances guided by objective activity data and existing guidelines, they did not explicitly integrate patient activity into their decision-making process. This suggests that health care practitioners may need further training and support on how to use objective measurements in a more explicit way when taking clinical decisions.

A further key finding was the lack of consistency in how wards described and discussed patient activity. No shared terminology existed to describe patient activity or functional status, creating a huge barrier for effective communication, and sharing of patient activity information. This aligns with a study by Hoyer et al.,^31^ which emphasized the importance of multidisciplinary communication, collaboration, and teamwork in transforming the culture of patient activity within hospitals. Establishing a common language is fundamental in this process, for example, using the same format or type of patient activity information, since a lack of shared terminology across wards can limit decision-making and ultimately affect care. Our findings reflected this heterogeneity: the same patient might be described as “active” in one ward but highly sedentary in another.

Another important point was the ownership of patient activity. Professional boundaries resulted in ambiguity regarding patient activity responsibilities. This ambiguity can result in delayed decision-making, as clinical decisions, such as timely calling a physiotherapist, changing the patient's position, or allowing the patient to walk independently, must be made at the appropriate moment. This lack of clarity in ownership leads to some patients having an insufficient level of activity that goes undetected and untreated.^19,32^ This lack of ownership has been shown to be caused by organizational barriers, hospital culture and lack of communication. Lack of timely clinical decisions can leave patients unattended, making them feel insecure and vulnerable when moving without assistance. As a result, they often engage in less physical activity.

Our findings raise important implications for clinical practice. Agreement on what type, amount, and quality of patient activity would help clinicians decide more precisely if a patient is ready for discharge or should be encouraged to move more to avoid iatrogenic complications.^33^ Developing more structured approaches to interpret patient activity, rather than relying mainly on intuition, could also reduce variability and support more consistent discharge planning.^34^ Together, these steps could help integrate patient activity more systematically into clinical decision-making and improve patient outcomes.

This study has several strengths. First, this study includes different health care practitioners from eight different hospital wards and various levels of seniority. Second, to reduce bias, interviews were conducted by researchers with different background, nursing, and physiotherapy. Finally, to strengthen the trustworthiness of the study, the final themes were shared with a number of participants after the analysis was completed. Their feedback indicated that the findings accurately reflected their experiences, which supports the credibility of the results. A qualitative design using observations and interviews was chosen to capture the hidden and often implicit processes underlying daily care decisions. This combination of observations and interviews is well established in qualitative health research, as it allows researchers to connect observed behaviors with practitioners’ reported reasoning and explanation.^25^

On the other side, this study has some limitations. First, most of the interviews were performed in English while the primary interviewer (CDM) had Spanish as native language and English level of C2, and all the interviewed participants had Dutch as their native language. However, in The Netherlands, 90–93% of the population have English proficiency. A minimum of B2 level is required after high school education. All the included participants had higher education. Therefore, we believe that language had a minor impact on our results. Second, results from this study are specific to a single hospital in The Netherlands. The limited generalizability of these results restricts their transferability to other clinical settings. Third, our observations and interviews focused primarily on the practitioners most directly responsible for patient activity in the hospital setting, namely, nurses, doctors, and physiotherapists. Including other professionals, such as social workers or occupational therapists, could have provided additional perspectives and enriched our understanding of how clinical decision-making around patient activity is shaped. Fourth and last, we acknowledge the possibility of an observer effect: participants may have modified their behavior during observations, appearing more rigorous or consistent in their practices because they were aware of being observed.

This study highlights how patient activity influences clinical decisions, such as discharge planning, medication, and surgery, and how, in turn, certain decisions can also impact patient activity. This study also reflects the complex interplay between mechanisms in the hospital setting and the ownership of patient activity. Future studies should focus on understanding explicit decision-making using patient activity information and the effects of improved multidisciplinary collaboration. The precise extent to which patient activity influences these clinical decisions is yet unknown.

## Clinical messages

- Patient activity influences clinical decisions, at the same time, clinical decisions influence patient activity.
- Roles for monitoring patient activity remain unclear; clearer responsibilities could improve consistency and teamwork in shared decision making.
- Clinicians often rely on intuition; incorporating objective measures of patient activity could support more reliable decisions.
- A shared language on patient activity is lacking; standardized terminology could strengthen communication and integration of patient activity into hospital care.

## Supplemental Material

sj-docx-1-cre-10.1177_02692155251393557 - Supplemental material for Exploring the role of patient activity in the clinical decision-making processes of health care practitioners working in hospital care: A qualitative study Supplemental material, sj-docx-1-cre-10.1177_02692155251393557 for Exploring the role of patient activity in the clinical decision-making processes of health care practitioners working in hospital care: A qualitative study by Carlos de Miguel Llorente, Miriam Van Der Velde, Ireen Scheffers, Cindy Veenhof and Karin Valkenet in Clinical Rehabilitation
