# Supplementary material for: Exploring the role of patient activity in the clinical decision-making processes of health care practitioners working in hospital care: A qualitative study
Source: Clin Rehabil. 2025 Nov 12;40(2):259–72. doi: 10.1177/02692155251393557 (PMC12816399; doi:10.1177/02692155251393557)
Supplement: sj-docx-1-cre-10.1177_02692155251393557 - Supplemental material for Exploring the role of patient activity in the clinical decision-making processes of health care practitioners working in hospital care: A qualitative study [file sj-docx-1-cre-10.1177_02692155251393557.docx]

**SUPPLEMENTARY 1**

**Data collection tool for clinical observations.**

| Setting/Ward | Date | Time start /stop | | ID | Observer | |
| --- | --- | --- | --- | --- | --- | --- |
| **Predetermined Items (Frequency of appearance)** | | | | | |  |
| Items | Context | | Comments | Frequency | |  |
| How many times does the HCP register patient activity information? |  | |  |  | |  |
| How many times does the HP asks about patient activity to their patients? |  | |  |  | |  |
| How many times does the HP ask about patient activity to their colleagues? |  | |  |  | |  |
| How many clinical decisions does the HR takes based on patient activity? |  | |  |  | |  |
| How often does the HP gives activity recommendations or guidelines to the patients? |  | |  |  | |  |
| **Open-ended descriptive fieldnotes:** | | | | | |  |

**SUPPLEMENTARY 2**

**Interview Guide Example**

| Domain | Topic | Subtopic |
| --- | --- | --- |
| Participant | Could you describe your background?  Could you explain how long you have been working as an HCP? And how long in the hospital setting?  What type of patients do you regularly see? |  |
| Introductory questions | Why do you think your role is important when it involves the physical activity and movement of the patient?  What do you think about patient activity during hospital stay? Do you consider is something you should oversee?  How do you observe and interpret patient activity during clinical encounters? |  |
| Decision | What role does patient activity play in your decision-making process?  How do you decide that a patient needs physiotherapy treatment?  Could you provide examples of how patient activity has helped you make a clinical decision in the past?  How has your approach to incorporating patient activity into decision-making evolved over your career? Do you consider it more important now than before? |  |
| Exchange | How do you exchange patient activity information with other HCP?  During interdisciplinary meetings, which HCP has the most important voice regarding patient activity? |  |
| Final | Do you take any other clinical decision based on patient activity? |  |

**SUPPLEMENTARY 3**

**Quality and Rigor.**

The research team consisted of five researchers. CDM and MVM were PhD candidates at University Medical Center Utrecht with professional backgrounds in physiotherapy. IS was a nurse and was enrolled in a master’s degree program at the time of the study. KV is an associate professor, and CV holds the position of lector. The team included both male and female researchers. CDM and IS conducted the interviews, while CDM, and KV carried out the observations. All team members had prior experience in qualitative research and engaged in ongoing reflexivity regarding their own roles and assumptions to strengthen the study’s analytical rigor.

Researchers of this study worked as hospital-researchers in the hospital setting where the study took place. The main researcher also had experience working as a physiotherapist in another hospital setting in the past. Hence, the researchers had previous knowledge on the patient activity area but lacked knowledge regarding health care practitioners’ clinical decision-making processes. Finally, the recruitment procedure was also influenced by the role of the researchers within the hospital. Participants were approached through face-to-face contact and email. They were informed about the study and invited to collaborate by voluntarily participating in interviews and/or observations. Physiotherapists were the first contacted as the working relationship with the main researcher was easier. Then, through them, nurses and doctors were contacted and recruited.

**SUPPLEMENTARY 4**

**Table 1**. Representative Participant Quotes Illustrating Each Subtheme Within the Main Themes.

| Themes | Subthemes | Participant Quotes |
| --- | --- | --- |
| 1. Patient activity influences decision making | Decisions on discharge destination | Doctors on discharge of a patient with low levels of activity but medically stable:  *“If a patient is internally stable, so cardiac is OK, without mechanical ventilation and can stay without mechanical ventilation and that patient won’t be doing any activity but the pressure on the beds is high…. they would push the patient out (of the hospital) and it will be OK...”* |
|  | Decisions on patient medication | Doctor on medication based on patients ‘activity:  *“We must give medication when patients are immobilized to prevent thrombosis….so that's the first thing, but that doesn't really depend on the physiotherapist. That's just if the patient is immobilized. So, we're giving him medication to prevent complications from staying in bed…”* |
|  | Decisions on walking aid | Doctor on nurses and physiotherapists deciding if a patient needs walking aid:  *“They see these patients all the time. I believe them more than myself, so that is why we also ask for their opinions during the multidisciplinary meetings, and they can say they are good in saying what is the current condition, strength, balance, and what advice do you have if using a walker or other things…”* |
|  | Decisions based on physical status | Doctor on clinical decisions based on physical status:  *“The cardiologist does the heart transplantations, the left ventricular assisted device, the little motors in the heart. There are quite strict guidelines when a patient is allowed to receive such treatment. Equal to the coronary heart bypass grafts and the big open-heart surgery. There are quite strict lines for transplantation and vascular surgery. So that's a bit outside of my grid. We see those patients sometimes before surgery to assess their frailty and see whether there are appropriate candidates for the intervention or the treatment. “* |
| 2. Clinical decision-making influences patient activity | Medication on patient activity | Physiotherapist in finding the balance between movement and medication:  “*So that's always the balance. Searching for the balance to not get them too much medication because the medication is not good for them as well. So, it's always if the level of medication, is appropriate or is it too much and it is affecting the patient.”* |
|  | Patient stimulation on patient activity | Nurse on promoting physical activity:  *“Yeah, I think it's a lot of reinforcement and eventually what happens is they start moving around, then it (the patient) moves a little bit, and they start feeling better and then it becomes a positive circle.”* |
|  | Hospital environment on patient activity | Nurse on changing the environment to improve patient activity:  *“An even more ideal situation would be that you just have your own practice space…So if you could set up something small here so that you and fellow patients have contact and movement. Then I think that's ideal.”* |
| 3.Multidisciplinary dynamics influence how patient activity is valued in clinical decisions | Multidisciplinary communication | Doctor on assessing and deciding together:  *“My clinical conversations are a lot about what kind of rehab someone needs. But sometimes I decide that another person needs to come and evaluate patients, so maybe if I see a problem with a patient who is on the cardiology, where to say, hey, let's call the neurologist to also evaluate if there's a neurological problem...”* |
|  | Facilitators for shared decision-making | Doctor on using information of nurses and physiotherapists to reach a decision:  *“I do see them (patients) myself. I use two things I read the report from the physiotherapist and from the nurses, so I see what they do in terms of physical and I sometimes verbally talk with them to confirm how easily this physical activity was and whether they needed support to move and if they needed support, how much that was...”* |
|  | Barriers for shared decision-making | Doctor on his role:  *“So, I think as a medical doctor, we're not that involved in the patient I think that on a daily basis the role is much more enhanced of the nurses and the physical therapists, or the occupational therapist… and our role is only to hear back from them on how the patient is moving...”* |
|  | Shared decision-making process | Doctor on assessing and deciding together:  *“…The earlier in admission, you know about the other things, the earlier you can also say what is the next best step for the patient after this admission. So, you can make sure the patient is not too long in the hospital, goes to the right place and you can treat new patients. Because there are more and more patients coming, so mobility I think is crucial, specially for the elderly. But I think maybe for everyone... That healthcare professionals should focus on in their decision-making. So, you must measure how secure is gait and quality of gait. What do you expect for the future? Are they goals? Can we train people? Do I need my physiotherapist to be more exact? What is the exact balance? What is the exact condition? What goals do you see for this patient? And then you make a combination of those things to make a good decision about the right treatment....”* |
| 4. Limited use of objective measurements influences how patient activity informs clinical decisions | Inconsistency of patient activity information | Doctor on lack of consistency of patient activity information:  *“I think in the future we will use more of those devices to see how much movement the patients get. And to make it more objective, yes…*  *So currently we don't really have it. We just stimulate and hope the patients get out of their beds and the nurses are important in that as well. But it's not like we are having thresholds or something, it's just something also we have in a quality agenda...”* |
|  | Objective assessment of patient activity | Nurse on using objective movement data:  “*Well, I do think it's a useful tool. That if I take it with me when reading it. Then I can see like, oh, this gentleman or lady has only walked five minutes today. Or has been active. Let's ask ourselves why that is and see if we can use it in the next few days.”* |

**SUPPLEMENTARY 5**

**Table 2**. Multidisciplinary Interactions in the ward, matters addressed, and health care practitioners involved.

| **Multidisciplinary Interaction** | **Matters addressed** | **Health Care Practitioners involved** |
| --- | --- | --- |
| Morning Visits | Overall Progress.  Daily Updates  Medication  Psychological status | Doctors  Nurses |
| Multidisciplinary meetings | Overall progress  Complex patients’ decisions  Discharge destination | Doctors  Nurses  Physiotherapists  Social workers  Rehabilitation Doctor |
| Physical encounters in the ward | Overall Progress  Changes in position of the patient  Motivation  Family involvement | Doctors  Nurses  Physiotherapists |
